# Interpretable polar-flow decomposition for torsional nystagmus lateralization in posterior-canal BPPV

**DOI:** 10.3389/fneur.2026.1963383

**Published:** 2026-10-02

**Authors:** Yanjun Li, Xiaomin Xu, Lidan Gao, Qiqi Shao, Jie Chen, Wenhuan Wang, Xiaoqing Li, Congcong Sun, Shike Hou, Xiaokai Yang

**Affiliations:** 1 School of Disaster and Emergency Medicine, Tianjin University, Tianjin, China; 2 Affiliated Wenzhou People’s Hospital of Hangzhou Medical College, Wenzhou People’s Hospital, Third Clinical College of Wenzhou Medical University, Wenzhou, Zhejiang, China

**Keywords:** benign paroxysmal positional vertigo, Dix–Hallpike maneuver, explainable artificial intelligence, nystagmus lateralization, optical flow, posterior semicircular canal, torsional nystagmus, vestibulo-ocular reflex

## Abstract

**Objective:**

Torsional nystagmus during the Dix–Hallpike maneuver represents a cardinal vestibulo-ocular reflex (VOR) sign in posterior-canal benign paroxysmal positional vertigo (pc-BPPV). This study develops an interpretable framework for automated classification of torsional nystagmus direction and temporal localization of abnormal events from clinical infrared recordings.

**Methods:**

Infrared Dix–Hallpike videos were processed via You Only Look Once version 8 nano (YOLOv8n) pupil localization, dynamic iris masking, and Recurrent All-Pairs Field Transforms (RAFT) optical flow. Flow vectors were projected into pupil-centered polar coordinates to extract tangential and radial motion features. A bidirectional gated recurrent unit with attention (BiGRU-attention) network performed segment-level three-class classification with sliding-window patient-level inference.

**Results:**

Five-fold cross-validation yielded segment-level accuracy of 94.19% ± 0.88% with balanced F1-scores across all three classes. On an independent 45-patient long-video test set, patient-level accuracy reached 97.78% at 60 frames per second (fps) and 100% (45/45) at 30 fps. Temporal localization achieved mean intersection-over-union (IoU) of 0.899 and 0.896, with hit rates of 96.8 and 100%, respectively.

**Conclusion:**

The polar-flow framework enables physiologically interpretable and robust classification of pc-BPPV-related torsional-upbeat nystagmus. By explicitly decomposing eye movements into tangential and radial components, it provides reliable lateralization while demonstrating the potential for clinically meaningful temporal localization of abnormal nystagmus events.

## 1 Introduction

Benign paroxysmal positional vertigo (BPPV) is the most common peripheral vestibular disorder affecting the semicircular canal-otolith system, with a lifetime prevalence of approximately 2.4% and a substantial tendency to recur (1, 2, 3, 4). Among its clinical subtypes, posterior-canal BPPV (pc-BPPV) accounts for the majority of cases, and its diagnosis relies heavily on the characteristic nystagmus elicited during positional testing (5, 6). In the Dix–Hallpike maneuver, pc-BPPV typically presents as mixed vertical-upbeating and torsional nystagmus, with the torsional direction providing essential information for lateralizing the affected ear (4, 5). Therefore, reliable characterization of torsional nystagmus, including its presence, rotational direction, and temporal evolution, is central to objective analysis of pc-BPPV-related eye movements.

From a vestibular physiology perspective, the diagnostic value of torsional nystagmus extends well beyond simple detection. The Dix–Hallpike maneuver is interpreted based on the presence of characteristic torsional upbeating nystagmus, while the rotational direction of the torsional component helps determine the affected side (5). Standardized characterization of nystagmus direction requires consideration of the relevant reference coordinate system (7). In addition, the duration and temporal profile of induced nystagmus provide useful clues for interpreting otoconial involvement, particularly in distinguishing canalithiasis from cupulolithiasis (8). An ideal automated tool must therefore not only detect torsional nystagmus but also robustly classify its direction and localize its onset and offset in long, unedited clinical videos.

Recent advances in deep learning have brought encouraging progress to video-based nystagmus analysis and BPPV diagnosis (9, 10, 11, 12, 13, 14, 15). Several studies have shown that models trained on video or eye-movement data can detect nystagmus, identify torsional patterns, and classify certain BPPV subtypes (10, 16, 17, 18). For example, Zhang et al. utilized a two-stream network for frame-level torsion recognition and segment localization, achieving an F1-score of 81.00%, though their method did not further distinguish torsional direction (19). Lim et al. projected iris texture onto polar coordinates and used inter-frame cross-correlation to quantify torsion, but the sensitivity dropped to 56.0% for right pc-BPPV compared with 88.2% for left pc-BPPV, revealing a marked imbalance in directional discrimination (20). Liu et al. combined scale-invariant feature transform (SIFT)-based iris tracking with fast Fourier transform (FFT) analysis to estimate torsional angular velocity and reported an 86.67% accuracy for rotational direction classification (11). More recently, Han et al. proposed a transformer-based system for torsional nystagmus detection and torsion-angle assessment, emphasizing the need for interpretable quantitative metrics in torsional eye-movement analysis (21). These traditional signal-processing pipelines, including cross-correlation, feature tracking, and frequency-domain analysis, may be vulnerable to clinical noise, eyelid occlusion, and inter-individual anatomical variation (22). More recently, Kong et al. used LiteFlowNet and ConvNeXt-LSTM for optical-flow-based classification and reached 97.75% accuracy for torsion presence detection; however, the per-class F1-scores for torsional subtypes in a four-class task fell to 0.822 and 0.735, indicating that stable and balanced differentiation of torsional direction remains an open challenge (23).

Notably, Qiu et al. recently introduced LLM-GAODE, a large-language-model-augmented framework that integrates a Gram-based attention mechanism with a neural ordinary differential equation network for video nystagmography (VNG) classification, achieving state-of-the-art performance on BPPV trajectory classification benchmarks (9). This work highlights the growing trend of incorporating advanced temporal modeling and interpretable reasoning into nystagmus analysis pipelines. Despite this progress, several methodological gaps persist that limit clinical translation in vestibular practice. First, most existing studies prioritize detecting the presence of torsion rather than achieving balanced left-versus-right lateralization, which is the clinically actionable question for guiding canalith repositioning therapy. Second, prevailing approaches often rely on whole-frame encoding or “black-box” spatiotemporal models that do not explicitly decouple torsional and linear eye movements in accordance with vestibulo-ocular reflex kinematics; this limits both physiological interpretability and robustness in the presence of artifacts and recording variability (24, 25, 26, 27). Third, clinical Dix–Hallpike recordings are typically long videos that encompass resting, positioning, nystagmus induction, and decay phases, yet systematic validation of patient-level inference strategies, such as temporal aggregation and long-video voting, remains scarce.

To address these gaps, we propose an interpretable deep-learning framework that explicitly decouples torsional and radial motion components in accordance with vestibulo-ocular reflex kinematics for automated pc-BPPV diagnosis using monocular infrared Dix–Hallpike videos. The framework employs YOLOv8n for robust pupil localization and constructs a dynamic iris-ring region of interest with an anatomical mask to suppress eyelid interference. Dense optical flow is estimated by RAFT and then projected into a pupil-centered polar coordinate system to derive separate tangential and radial velocity features that directly represent the pathognomonic torsional and linear eye movements. A bidirectional gated recurrent unit with attention (BiGRU-attention) network classifies short segments, and a sliding-window aggregation strategy with confidence-based filtering is introduced for reliable patient-level inference from full-length recordings. By design, this explicit polar-flow decomposition aligns the computational model’s decision logic with established vestibular physiology and provides inherent interpretability. Extensive experiments on a clinical dataset, including an independent long-video test set, confirm that the framework achieves balanced lateralization and high resilience even under suboptimal recording conditions, highlighting its potential for translation into real-world neurotology practice.

## 2 Methods

### 2.1 Overall framework

All videos were acquired during the Dix–Hallpike maneuver and were clinically reviewed according to the positional nystagmus pattern. In accordance with the diagnostic criteria for posterior-canal BPPV, a positive pc-BPPV reference label required the presence of characteristic torsional nystagmus accompanied by an upbeat vertical component. The affected side was determined according to the direction of the torsional quick phase. Following the Bárány Society nomenclature, clinical torsional direction was described in head-referenced terms according to the direction in which the upper pole of the eye beat toward the right or left ear (7). Typical posterior-canal BPPV was identified by torsional-upbeat positional nystagmus, with the upper pole beating toward the dependent ear. Accordingly, a response elicited during the right Dix–Hallpike maneuver with the upper pole beating toward the right ear was classified as right posterior-canal BPPV, whereas the corresponding pattern during the left Dix–Hallpike maneuver was classified as left posterior-canal BPPV (7, 28). The normal-negative category was defined as recordings in which the Dix–Hallpike maneuver did not elicit the characteristic torsional-upbeat response of pc-BPPV or any other clearly position-evoked nystagmus pattern. Background spontaneous nystagmus was not used as an exclusion criterion. Therefore, recordings showing isolated torsional, horizontal, downbeat, torsional-downbeat, or other atypical positional nystagmus patterns were not assigned to the normal-negative category and were excluded from the present three-class classification task. Throughout this study, the direction of torsional nystagmus refers exclusively to the quick-phase direction. The oppositely directed slow phase was not used to determine the class label or affected laterality.

All video-level classifications and temporal ground-truth annotations were independently performed by two clinicians experienced in vestibular disorders. Any discrepancies were first resolved through joint review and discussion. If disagreement persisted, a third senior reviewer participated in the adjudication, and the final reference annotation was established by consensus. For each video, the nystagmus category, affected laterality, and the onset and offset of nystagmus episodes were determined based on the visible torsional-upbeat pattern, with torsional direction defined according to the quick phase.

We developed an automated analysis framework for infrared monocular videos acquired during the Dix–Hallpike maneuver to differentiate left posterior-canal BPPV, right posterior-canal BPPV, and normal-negative cases. The pipeline consisted of five sequential modules: (1) pupil localization using You Only Look Once version 8 nano (YOLOv8n); (2) dynamic iris region reconstruction with an anatomical mask; (3) dense optical flow estimation and polar coordinate motion decomposition; (4) segment-level temporal classification using a BiGRU-attention network; and (5) patient-level long-video inference. The overall workflow is illustrated in Figure 1. This study was a retrospective analysis of clinical video data collected between 2020 and 2023. The study protocol was reviewed and approved by the Ethics Committee of Wenzhou People’s Hospital (Approval Number: KY-2023-190). All procedures were conducted in accordance with the ethical standards of the 1964 Declaration of Helsinki and its later amendments.

**Figure 1:**
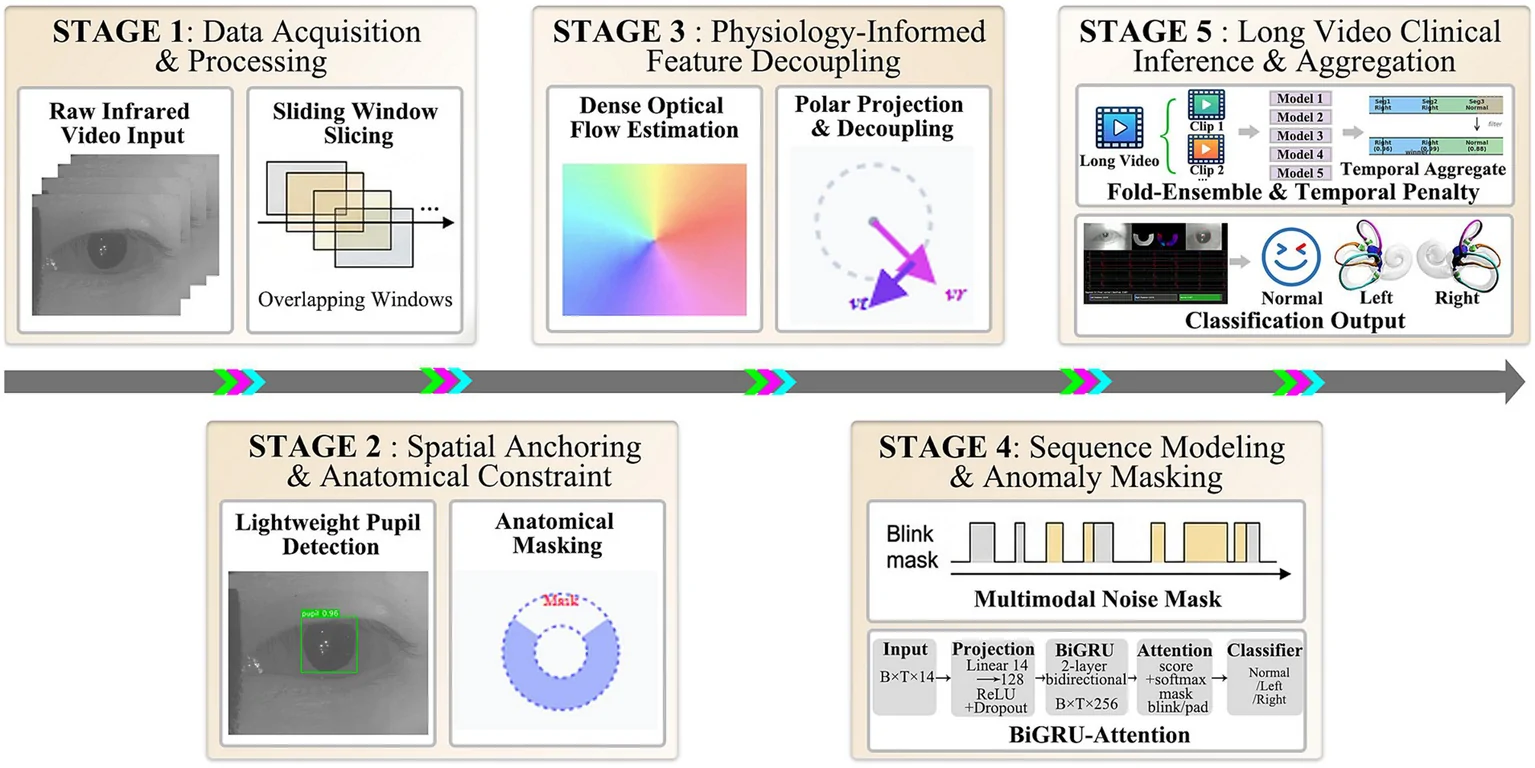
Overall workflow of the proposed polar-flow decomposition framework for torsional nystagmus lateralization in posterior-canal BPPV.

### 2.2 Dataset construction and cross-validation

Infrared eye-movement videos were acquired during the Dix–Hallpike maneuver using the G-Force Vertigo Diagnosis and Treatment System (Guoyi Huake [Tianjin] Medical Technology Group Co., Ltd., Tianjin, China). The recordings analyzed in the present study consisted of monocular left-eye grayscale videos acquired at a spatial resolution of 640 × 360 pixels and a sampling rate of 60 frames per second. To standardize temporal modeling, each long video was segmented using a sliding window of 5 s with a stride of 2.5 s, resulting in clips of approximately 300 frames. Because posterior-canal BPPV exhibits a mirror-symmetric directional pattern between the left and right sides, all samples were additionally subjected to horizontal-flip augmentation with simultaneous label swapping.

The final classification dataset comprised 262 independent subjects (Supplementary Table S1): 82 with left posterior-canal BPPV, 83 with right posterior-canal BPPV, and 97 originally normal-negative subjects. Among the 165 BPPV subjects, 81 contributed additional posttreatment negative recordings after successful canalith repositioning, bringing the total normal-negative segments to 2,965. Because this was a retrospective study, post-repositioning recordings were obtained from patients who had already been clinically judged to have undergone successful canalith repositioning before dataset construction. These follow-up recordings were included in the normal-negative class when repeat Dix–Hallpike testing no longer elicited the characteristic torsional-upbeat positional nystagmus of posterior-canal BPPV. Spontaneous nystagmus, including horizontal spontaneous nystagmus, was not used as an exclusion criterion; however, recordings showing other clearly position-evoked nystagmus patterns were excluded from the negative class. Inclusion of these post-repositioning normal-negative recordings was intended to reduce potential confounding from subject-specific ocular appearance and other non-pc-BPPV visual characteristics, thereby encouraging the model to focus on the dynamic positional eye-movement pattern relevant to pc-BPPV. Because positional nystagmus in pc-BPPV may exhibit fatigability with repeated provocation, repeated Dix–Hallpike examinations were separated by a resting interval of at least 15 min and were performed only after symptoms elicited by the preceding examination had subsided (7).

To prevent data leakage, this study strictly implemented a patient-level nested 5-fold cross-validation protocol, ensuring that data from any single patient never spanned across different subsets. When follow-up negative recordings belonged to patients who also had pre-repositioning pathological recordings, all recordings from that patient were kept within the same data split to preserve strict subject-level independence. In the outer loop, all patients were partitioned into five mutually independent subsets for unbiased performance evaluation. Within each fold, an internal validation set was separated from the training data at a 15% ratio (the inner loop) to facilitate hyperparameter selection and early stopping control. The detailed distribution of training, validation, and test samples across the five folds is provided in Supplementary Table S2.

### 2.3 Pupil anchoring and iris region-of-interest reconstruction

To obtain a stable spatial reference, a dedicated pupil-detection model was trained using an auxiliary dataset constructed from 100 independent normal subjects who were not included in the main classification task. Frames were sampled at 5-s intervals. Annotation followed a semi-automatic procedure: contrast was first enhanced using contrast-limited adaptive histogram equalization, pseudo-labels were then generated by combining Hough circle detection and Otsu thresholding, and all bounding boxes were finally corrected manually. A lightweight YOLOv8n detector was trained for 100 epochs with an input size of 640 × 640 and standard augmentation, including Mosaic. During inference, a confidence threshold of 0.5 was used; frames below this threshold were treated as missed detections or invalid frames.

Within each 5-s clip, pupil localization was performed frame-by-frame, and the median pupil-center coordinates of all valid detections were used as the global spatial anchor. The region of interest (ROI) size was determined dynamically from the maximum pupil radius observed across these valid frames together with an outer-ring scale factor and a safety margin, and the local ROI was normalized to 256 × 192 pixels by isotropic enlargement followed by centered cropping. Clips with fewer than 50% valid frames were discarded because they could not be anchored reliably.

A double-ring concentric mask was then constructed around the pupil center, with an inner radius of 1.05R and an outer radius of 2.0R, where R denotes the current pupil radius. This ring defined the main analysis region containing the most informative iris texture. To suppress eyelid- and eyelash-induced pseudo-motion, pixels located in the superior angular sector from −165° to −15° in polar coordinates were further excluded.

### 2.4 Optical-flow features and sequence quality control

After ROI normalization, dense optical flow between adjacent frames was estimated using Recurrent All-Pairs Field Transforms (RAFT). To improve temporal stability, features were computed only on the intersection of the valid masks from two consecutive frames, followed by a 3 × 3 morphological erosion to reduce boundary artifacts.

To explicitly characterize torsional and linear motion patterns, the optical-flow field F = (u, v) was projected onto a local polar coordinate system centered at the pupil. For a pixel with relative position d = (dx, dy), tangential and radial velocities were defined as follows:

$$\mathrm{vt}=\frac{v\;\mathrm{dx}-u\;\mathrm{dy}}{(\sqrt{dx^{2}+dy^{2}+\varepsilon )}}$$ (1)

$$\mathrm{vr}=\frac{\left(u\;\mathrm{dx}+v\;\mathrm{dy}\right)}{\left(\sqrt{dx^{2}+dy^{2}+\varepsilon }\right)}$$ (2)

The sign of the tangential-flow component was defined in the video image coordinate system for computational purposes only, whereas clinical laterality labels followed the head-referenced Bárány Society terminology described above (28).

For each adjacent frame pair, a 14-dimensional feature vector was extracted from the valid iris-ring region, including the mean and standard deviation of horizontal and vertical flow components (*vx*, *vy*), the mean, standard deviation, and maximum of flow magnitude, the mean and standard deviation of tangential and radial velocities, and three geometric context variables, namely the smoothed pupil-center coordinates (*xc*, *yc*) and pupil radius R (The detailed definitions are provided in Supplementary Table S3).

To control blink- and artifact-related corruption, any frame pair containing a missed pupil detection in either frame was initially marked invalid. To account for temporal spillover of blink-related deformation, invalid labels were extended by two frames before and after each original invalid position to generate a blink mask. This mask was used in two ways: Z-score normalization statistics were calculated only from valid non-blink frames, and invalid time steps were explicitly masked in the attention module during temporal modeling.

### 2.5 Temporal classifier and training strategy

The primary classifier was a BiGRU-attention network (Figure 2). At each time step, the 14-dimensional feature vector was mapped to a 128-dimensional hidden space, followed by ReLU activation and dropout. The resulting sequence was then processed by a two-layer bidirectional GRU, yielding a 256-dimensional concatenated hidden representation. A temporal attention module was subsequently applied to assign larger weights to diagnostically informative time steps, and the attention-weighted sequence representation was passed to a fully connected head to obtain three-class probabilities.

**Figure 2:**
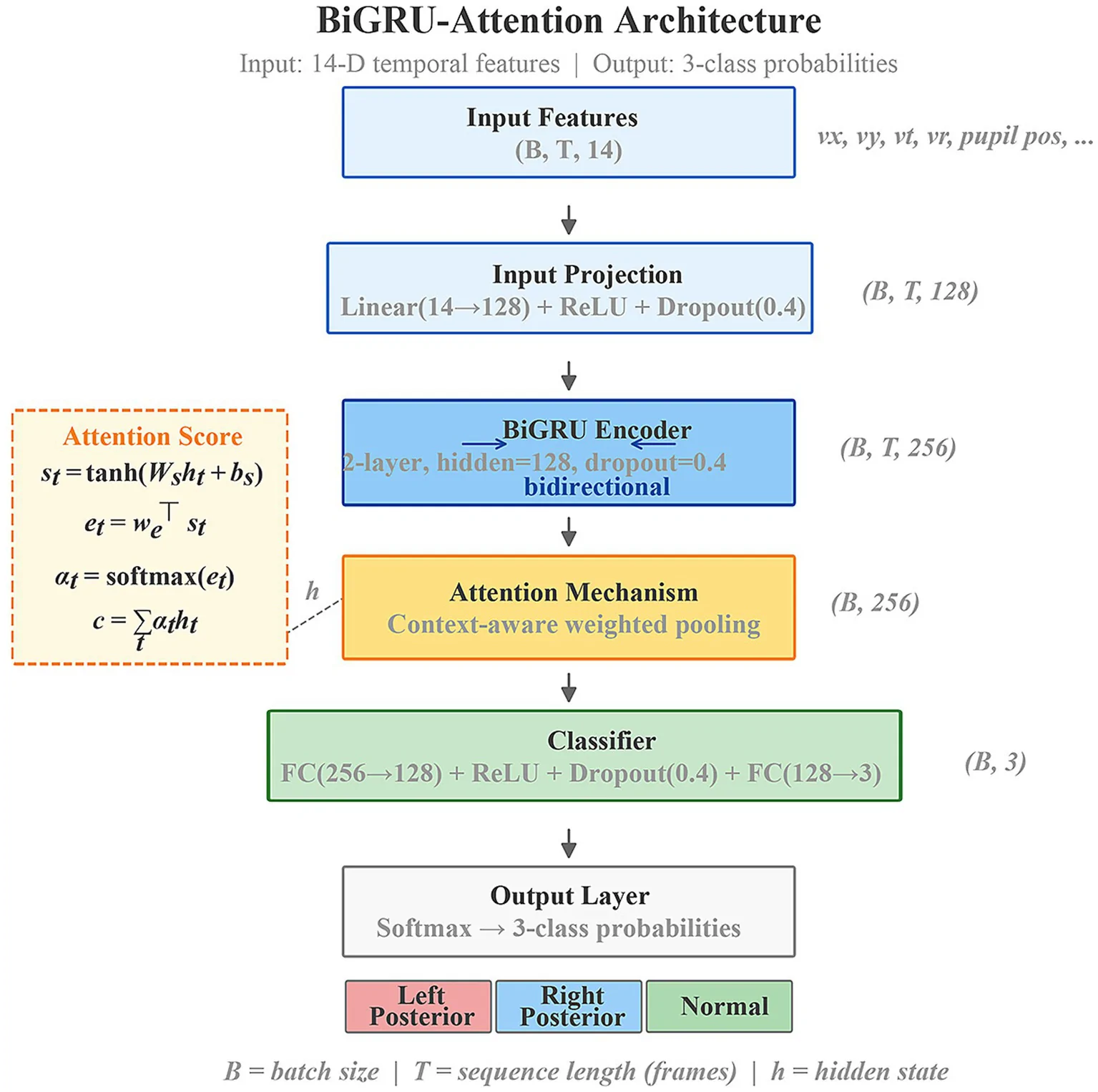
Architecture of the BiGRU-attention network for segment-level temporal classification.

Training used the AdamW optimizer with an initial learning rate of 1 × 10^−3^, weight decay of 1 × 10^−4^, batch size of 64, and a maximum of 100 epochs (Supplementary Table S4). Gradient clipping was set to 1.0. Learning-rate decay was controlled by ReduceLROnPlateau with a patience of 5 epochs and a decay factor of 0.5, whereas early stopping used a patience of 15 epochs. To alleviate class imbalance and improve optimization stability, we employed a WeightedRandomSampler and label smoothing of 0.05. All experiments were run with a fixed random seed of 42.

Under the same training protocol, a comparison was made among three temporal encoders, specifically BiGRU, bidirectional long short-term memory (BiLSTM), and Transformer. Additional sensitivity analyses were performed with respect to RAFT iteration number (3, 6, and 12 iterations), frame rate (original 60 fps vs. downsampled 30 fps), and the contribution of horizontal-flip augmentation.

### 2.6 Long-video inference and patient-level aggregation

To extend the segment-level model to full Dix–Hallpike recordings, long videos were processed using a patient-level inference workflow. First, the feature sequence of each long video was segmented with a 300-frame sliding window and 50% overlap. Each segment was then evaluated by the five models obtained from the outer cross-validation folds, and the predicted probabilities were averaged to reduce variance and improve robustness. For inference on the independent long-video test set, we ensembled the five BiGRU models derived from the patient-level cross-validation procedure, all of which were trained exclusively on the development dataset and had no overlap with the independent test subjects.

A two-stage filtering strategy was used to remove unreliable segments. Segments were discarded if the invalid-frame ratio was ≥30% or if the maximum ensembled class probability was <55%. In addition, to suppress weak abnormal predictions arising during the decay phase of nystagmus, a temporal tail-downweighting rule was introduced. Once a sustained high-confidence abnormal event had been detected, subsequent suspicious abnormal segments embedded in a predominantly normal tail region were penalized by reducing their voting weight to 0.35. The remaining qualified segments entered the final weighted-majority vote for patient-level classification. Adjacent abnormal segments with the same class label were merged into single events, and their onset, offset, and mean confidence were recorded. The complete pipeline for this temporal aggregation and voting strategy is illustrated in Figure 3. This tail-downweighting rule was used solely as a post-processing heuristic to suppress low-confidence predictions during the decay phase and was not intended to exclude prolonged, low-amplitude nystagmus from clinical consideration.

**Figure 3:**
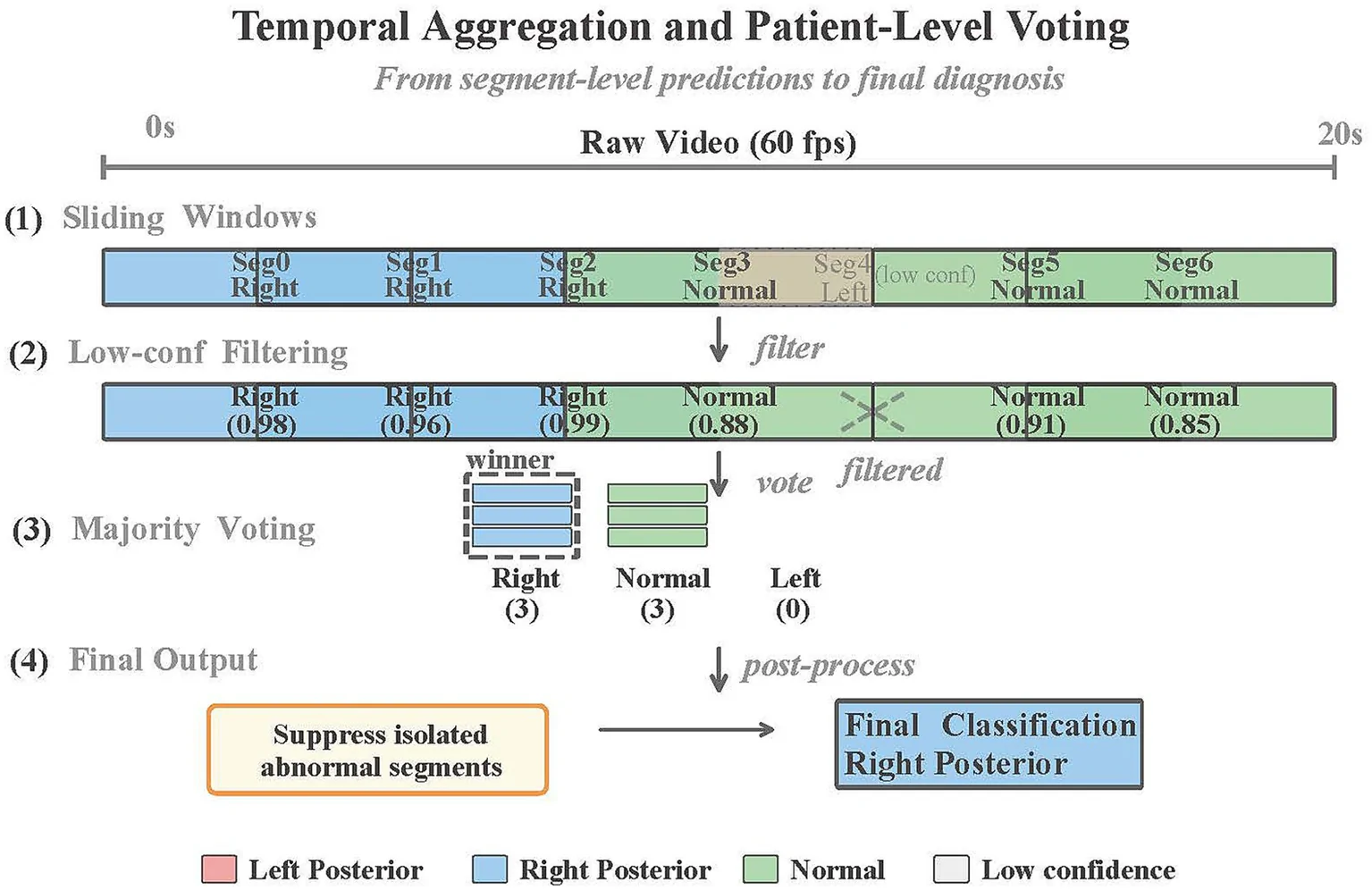
Workflow of the temporal aggregation and patient-level voting strategy. (1) Sliding window slicing of the raw long video and segment-level classification using the 5-fold model ensemble. (2) Unreliable segments are removed through low-confidence filtering. (3) A majority voting mechanism is applied to the remaining valid segments to aggregate temporal predictions. (4) The final patient-level classification is generated after suppressing isolated abnormal segments.

### 2.7 Independent long-video test set

To assess generalizability under conditions closer to real clinical workflow, an independent patient-level long-video test set was additionally established. The independent long-video test set was not used for model training, validation, hyperparameter tuning, threshold selection, or post-processing rule development. It contained 45 Dix–Hallpike-related long videos from different patients, including 14 normal videos, 14 left posterior-canal BPPV videos, and 17 right posterior-canal BPPV videos. The set intentionally included challenging cases, such as recordings with short-lasting nystagmus, frequent blinking, and changes in recording angle (representative frames are shown in Supplementary Figure S1). For each video, segment-level probability curves, class trajectories, and temporal profiles of blink ratio and model confidence were generated for diagnostic review and abnormal-event localization.

### 2.8 Implementation details

All experiments were conducted on Ubuntu 24.04.4 LTS with an Intel Core i9-14900KF CPU, 64 GB RAM, and an NVIDIA GeForce RTX 3090 GPU. The framework was implemented in Python 3.13.5 and PyTorch 2.6.0 with CUDA 12.1. The random seed was fixed at 42, and deterministic CuDNN behavior was enabled.

### 2.9 Evaluation metrics and statistical analysis

Model performance was evaluated at the segment, patient, and temporal-event levels. For segment-level three-class classification, overall accuracy was calculated as the proportion of correctly classified segments. For each class, precision, recall, and F1-score were calculated using a one-versus-rest strategy, where precision = TP/(TP + FP), recall = TP/(TP + FN), and F1-score = 2 × precision × recall/(precision + recall). Here, TP, FP, and FN denote true positives, false positives, and false negatives for the target class, respectively. Macro-averaged metrics were obtained by averaging the class-wise values across the three diagnostic categories. Receiver operating characteristic area under the curve (ROC AUC) was also calculated for each class using a one-versus-rest approach.

For independent long-video inference, patient-level predictions were obtained using the predefined temporal aggregation strategy, including quality filtering, confidence filtering, tail downweighting, and weighted-majority voting. Patient-level accuracy was defined as the proportion of correctly classified long videos. Specificity was calculated for normal-negative cases, whereas class-specific recall was calculated for left and right posterior-canal BPPV cases.

For videos with ground-truth (GT) temporal annotations, the agreement between predicted and annotated nystagmus intervals was assessed using temporal intersection-over-union (IoU), hit rate, and duration ratio. For each video and each laterality class, all GT intervals of the same class were merged into a single temporal set, and all predicted intervals of the same class were merged similarly. Video-level temporal IoU was computed as the ratio of the intersection duration to the union duration between the predicted and GT temporal sets. If both sets were empty, IoU was set to 1; if only one set was empty, IoU was set to 0. Hit rate was defined as the proportion of annotated BPPV videos in which at least one correctly lateralized predicted interval temporally overlapped with the GT interval. The duration ratio was calculated as the total predicted event duration divided by the total GT event duration for each matched laterality class.

## 3 Results

### 3.1 Performance of spatial anchoring and feature extraction

After 100 epochs, the model converged rapidly and achieved a precision of 99.99%, a recall of 100.00%, and a mean average precision at an IoU threshold of 0.50 (mAP@50) of 99.50% at a confidence threshold of 0.5; the detailed training dynamics and operating curves are provided in Supplementary Figure S2. These findings indicate that the detector provided reliable pupil localization under challenging clinical conditions, including infrared reflections, variable gaze directions, and partial eyelash occlusion, thereby establishing a consistent spatial reference for the subsequent processing steps.

A comprehensive step-by-step visual demonstration of this pipeline is presented in Figure 4. Starting from a raw clinical infrared video frame (Figure 4A), the YOLOv8n detector achieves high-precision pupil localization and dynamic iris-ring reconstruction (Figure 4B). This structural reference enables precise ROI cropping and the application of an anatomical mask to effectively block upper eyelid and eyelash interference (Figure 4C). Based on this clean masked region, dense optical flow is reliably estimated using RAFT (Figure 4D), which is ultimately transformed into polar coordinates to extract a 14-dimensional spatiotemporal feature sequence (Figure 4E). This explicit decoupling of torsional (*vt*) and linear (*vr*) motion components forms the basis for accurate downstream temporal classification.

**Figure 4:**
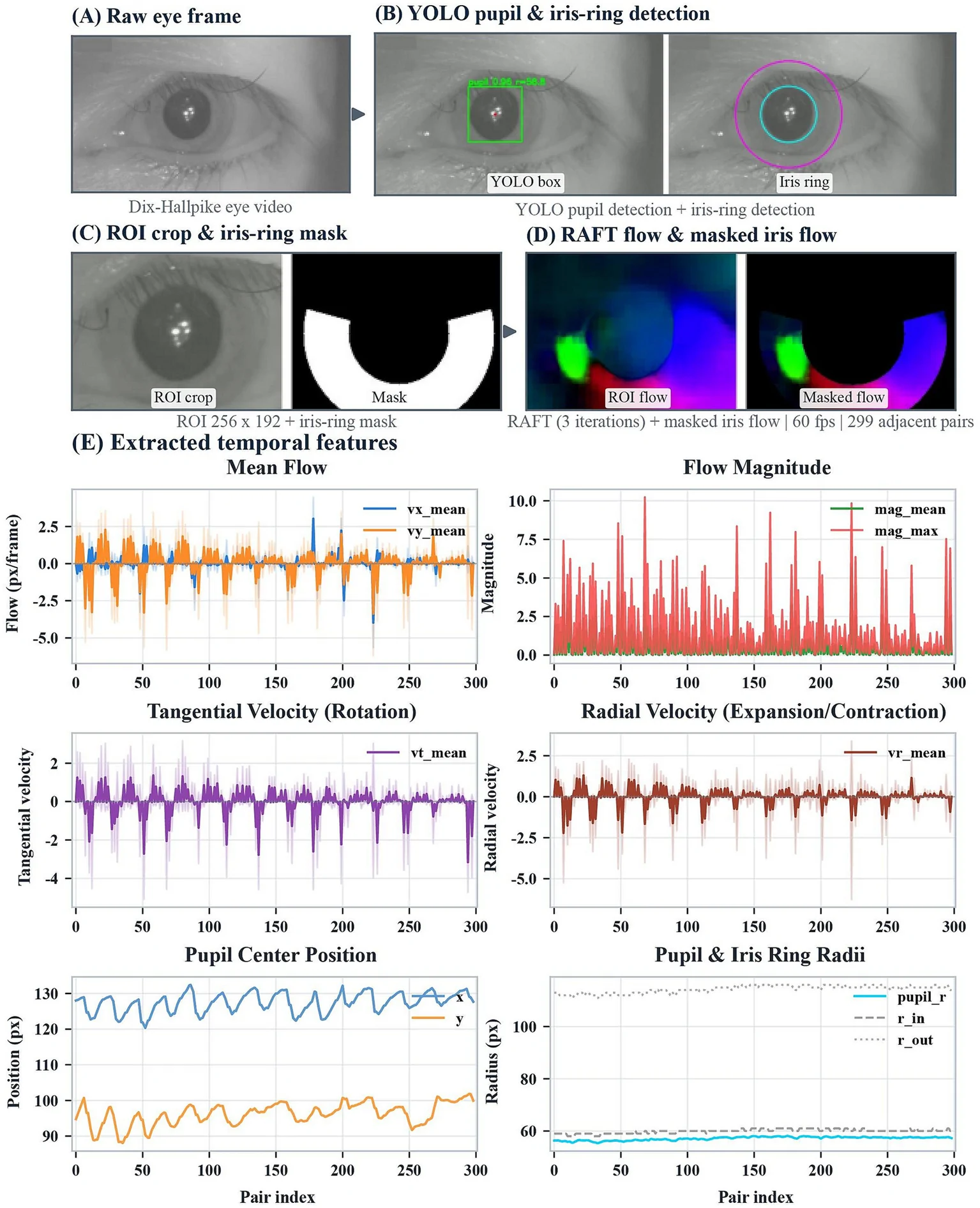
Pipeline for spatial anchoring and spatiotemporal feature decoupling. **(A)** Raw clinical infrared nystagmus video frame. **(B)** High-precision pupil center localization and dynamic iris ring (outer and inner rings) reconstruction using a lightweight YOLOv8n. **(C)** Cropped fixed-size region of interest (ROI) and the anatomical mask designed to block upper eyelid and eyelash interference (−165° to −15°). **(D)** Dense optical flow field estimated by a lightweight RAFT algorithm, alongside the visualization of the masked effective flow field. **(E)** The extracted 14-dimensional spatiotemporal feature sequence, including the mean and magnitude of translational flow, tangential torsional velocity (vt) and radial velocity (vr), and geometric context parameters (pupil position and radius), enabling the explicit decoupling of torsional and linear motions.

### 3.2 Segment-level three-class classification

#### 3.2.1 Overall comparison of sequence encoders

Using the same data split, feature representation, and training protocol, we compared three sequence encoders: BiGRU, BiLSTM, and Transformer (Table 1). Across the five outer folds, BiGRU achieved the best overall performance and the smallest fold-to-fold variation, with a mean accuracy of 94.19% ± 0.88%, precision of 94.30% ± 0.65%, recall of 93.80% ± 1.08%, and F1-score of 93.99% ± 0.81%. The corresponding mean F1-scores for BiLSTM and Transformer were 93.67% ± 1.40 and 89.93% ± 2.41%, respectively. The validation results followed the same trend; accordingly, BiGRU was selected as the primary model for the subsequent analyses and patient-level inference. The detailed fold-to-fold performance metrics of the three models are visualized in Figure 5, providing a comprehensive comparison across multiple dimensions. Specifically, the BiGRU model consistently outperformed the BiLSTM and Transformer architectures in terms of Macro F1-score (Figure 5A) and overall Accuracy (Figure 5B) across all five folds. This superiority was also maintained in Macro Precision (Figure 5C) and Macro Recall (Figure 5D), where the BiGRU displayed the smallest variance, further validating its robustness as the optimal sequence encoder for this task.

**Table 1:** Overall test-set performance comparison among BiGRU, BiLSTM, and Transformer.

Model	Accuracy (%)	Precision (%)	Recall (%)	F1-score (%)
BiGRU	**94.19 ± 0.88**	**94.30 ± 0.65**	**93.80 ± 1.08**	**93.99 ± 0.81**
BiLSTM	93.85 ± 1.36	93.90 ± 1.24	93.52 ± 1.62	93.67 ± 1.40
Transformer	90.07 ± 2.23	89.68 ± 2.11	90.89 ± 3.14	89.93 ± 2.41

Values are presented as mean ± standard deviation (SD) across the five outer folds. Bold values indicate the best performance in each metric.

**Figure 5:**
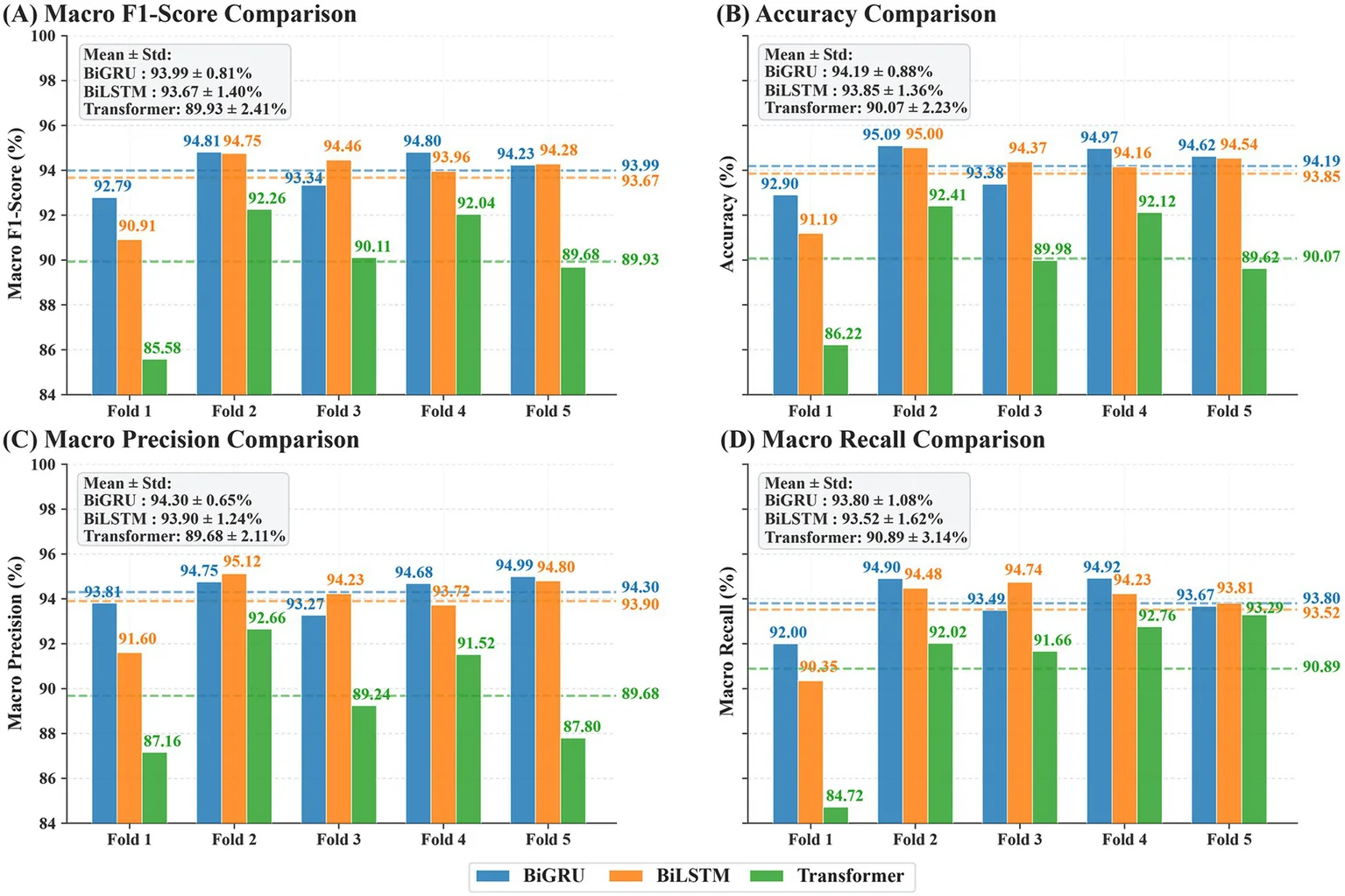
5-fold cross-validation performance comparison of different sequence encoders (BiGRU, BiLSTM, and Transformer) in the segment-level three-class classification task. **(A–D)** Macro F1-score, Accuracy, Macro Precision, and Macro Recall, respectively, across the 5 folds for the three models. The horizontal dashed lines with error bars represent the mean performance and standard deviation for each model.

#### 3.2.2 Class-wise performance of the final BiGRU model

In the final BiGRU model, all three classes were recognized with good balance (Table 2). The F1-scores for Normal, Left Posterior, and Right Posterior were 94.87% ± 1.23, 93.73% ± 1.05, and 93.37% ± 0.94%, respectively, and the receiver operating characteristic area under the curve (ROC AUC) values remained above 0.980 for all classes. Notably, the recall of the Right Posterior class was slightly lower than that of the other two classes (92.23% ± 3.52%), suggesting that this category was relatively more challenging in weak or short-duration abnormal segments.

**Table 2:** Class-wise recognition performance of the final BiGRU model.

Class	Precision (%)	Recall (%)	F1-score (%)	ROC AUC
Left posterior	93.87 ± 1.31	93.65 ± 2.40	93.73 ± 1.05	0.980 ± 0.010
Right posterior	94.73 ± 2.50	92.23 ± 3.52	93.37 ± 0.94	0.980 ± 0.009
Normal	94.30 ± 2.41	95.51 ± 1.44	94.87 ± 1.23	0.984 ± 0.008

### 3.3 Effectiveness of spatiotemporal feature decoupling and sensitivity analyses

#### 3.3.1 Effect of horizontal flip augmentation

To verify the value of anatomically informed augmentation, we compared models trained on the original dataset alone with those trained on the original data plus horizontally flipped samples (Table 3). The addition of mirrored samples increased the mean test F1-score from 91.59% ± 2.02 to 93.99% ± 0.81%, corresponding to an absolute gain of 2.40 percentage points. Mean accuracy also improved from 92.13% ± 2.02 to 94.19% ± 0.89%. The reduction in fold-to-fold variability further suggests that mirror-based augmentation improved both performance and model stability.

**Table 3:** Combined ablation study across RAFT iterations, frame rate, and flip augmentation.

Setting	Accuracy (%)	Precision (%)	Recall (%)	F1-score (%)	ΔF1 vs best (pp)
3-iter (60 fps, Flip)	**94.19 ± 0.89**	**94.30 ± 0.65**	93.80 ± 1.08	**93.99 ± 0.81**	0.00
6-iter	94.03 ± 1.07	93.96 ± 0.97	**93.88 ± 1.23**	93.89 ± 1.04	−0.10
12-iter	93.46 ± 1.30	93.37 ± 1.03	93.29 ± 1.60	93.30 ± 1.32	−0.69
30 fps	94.03 ± 1.59	94.25 ± 0.88	93.61 ± 2.04	93.86 ± 1.54	−0.13
Original-Only	92.13 ± 2.02	92.00 ± 2.53	91.54 ± 1.61	91.59 ± 2.02	−2.40

Values are presented as mean ± SD across the five outer folds. pp denotes percentage points. ΔF1 vs best is computed within each ablation factor relative to the highest F1-score in that factor. Bold values indicate the best Accuracy, Precision, Recall, and F1-score within each ablation group.

At the class level, the gains were more pronounced for the pathological classes: the F1-scores of Left Posterior and Right Posterior increased by 3.62 and 2.38 percentage points, respectively, whereas the Normal class improved by 1.20 percentage points. These findings indicate that left–right symmetry-based augmentation effectively enriched directional nystagmus patterns and improved learning of side-specific cues.

#### 3.3.2 Effect of the number of RAFT iterations

To determine an efficient optical-flow configuration, we compared features extracted using 3, 6, and 12 RAFT iterations (Table 3). Three iterations were sufficient for the current task and yielded the highest mean test F1-score (93.99% ± 0.81%) and accuracy (94.19% ± 0.89%). Increasing the number of iterations to 6 and 12 reduced the mean F1-score to 93.89% ± 1.04 and 93.30% ± 1.32%, respectively.

In terms of computational cost, processing a 300-frame cropped ROI sequence required approximately 1.63 s with 3 iterations, 2.39 s with 6 iterations, and 4.00 s with 12 iterations. Taken together, these results indicate that 3 iterations provided the best balance between computational efficiency and diagnostic performance.

#### 3.3.3 Frame-rate sensitivity analysis

Under the same data split and training configuration, we further compared the original 60 fps feature sequences with those downsampled to 30 fps. The overall classification performance remained essentially unchanged, as illustrated by the nearly identical Receiver Operating Characteristic (ROC) and Precision-Recall (PR) curves between the 60 fps (Figures 6A,B) and 30 fps (Figures 6D,E) models. The mean F1-score was 93.99% ± 0.81% at 60 fps and 93.86% ± 1.54% at 30 fps, a difference of only 0.13 percentage points (Table 3). No systematic decline was observed in the class-wise F1-scores, which is further supported by the highly consistent aggregated confusion matrices for both the 60 fps (Figure 6C) and 30 fps (Figure 6F) configurations.

**Figure 6:**
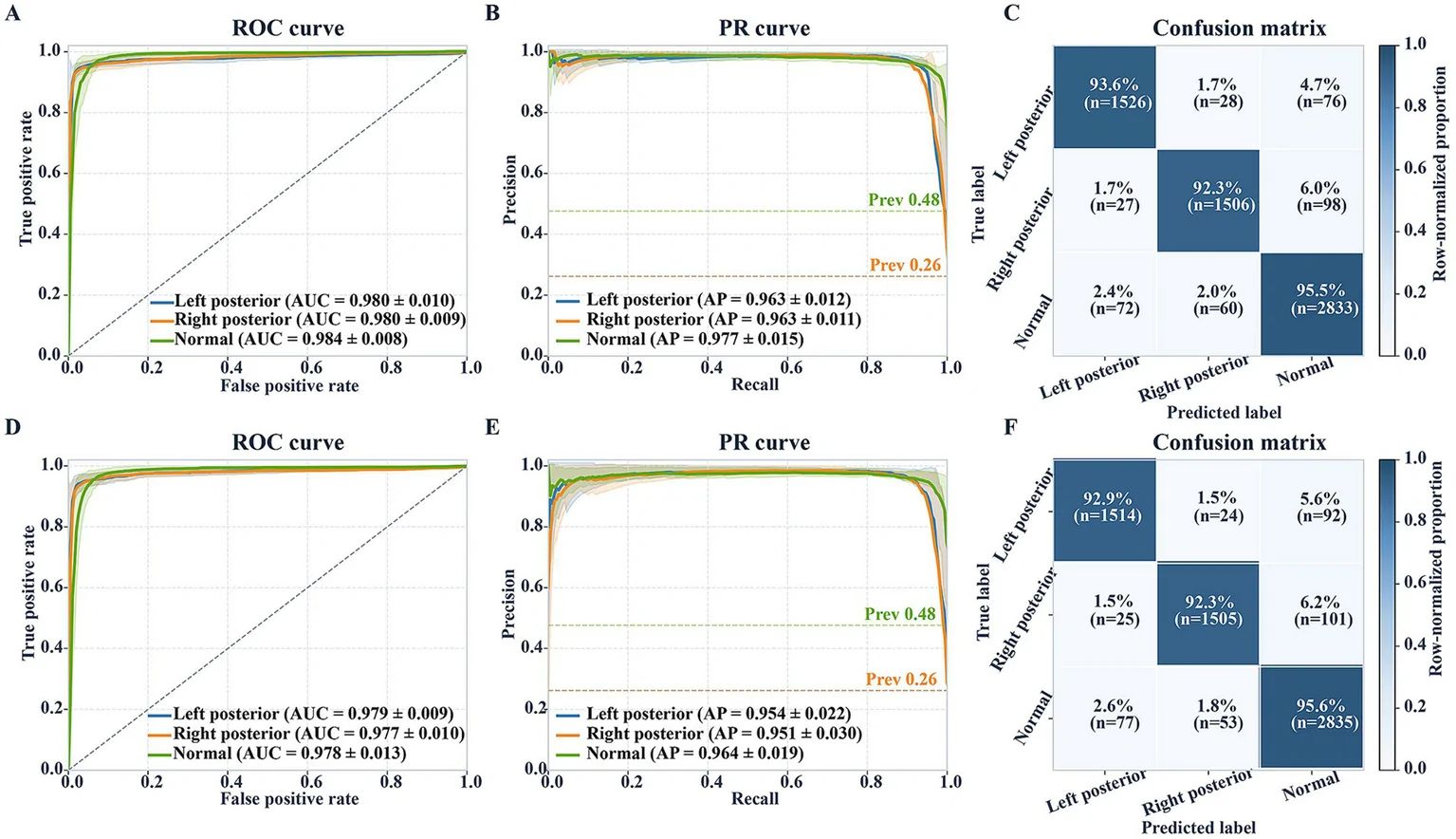
Classification performance evaluation of the 60 fps and downsampled 30 fps models under 5-fold cross-validation. **(A–C)** Receiver Operating Characteristic (ROC) curves, Precision-Recall (PR) curves, and aggregated class-wise confusion matrix for the 60 fps model. **(D–F)** The corresponding curves and confusion matrix for the 30 fps downsampled model.

These findings suggest that the dominant temporal patterns of posterior-canal BPPV during the Dix–Hallpike maneuver could already be captured at 30 fps, and that the additional temporal redundancy at 60 fps did not materially improve discrimination. From a deployment perspective, the 30 fps configuration may reduce computational burden while maintaining comparable performance in the present dataset.

#### 3.3.4 Ablation of polar-motion components

To quantify the contribution of polar motion decomposition, we performed a feature ablation study using the BiGRU model. As illustrated in Table 4, when only the four polar-motion features were retained, the model still achieved an F1-score of 93.57% ± 0.88%, only 0.42 percentage points lower than the full-feature model. In contrast, using only the seven basic Cartesian optical-flow features reduced the F1-score to 86.70% ± 0.97%, and using only the three geometric features further reduced it to 62.62% ± 4.14%.

**Table 4:** Overall classification performance of different feature subsets.

Feature subset	Dimensionality	Accuracy (%)	Precision (%)	F1-Score (%)
Full feature set	14D	94.19 ± 0.88	94.30 ± 0.65	93.99 ± 0.81
Polar-motion only	4D	93.73 ± 0.88	93.80 ± 0.97	93.57 ± 0.88
Basic optical flow only	7D	88.27 ± 1.06	87.10 ± 1.27	86.70 ± 0.97
Geometric features only	3D	67.58 ± 4.07	64.71 ± 3.32	62.62 ± 4.14

At the class level, the model using only the basic flow features achieved F1-scores of 82.91 and 83.32% for Left Posterior and Right Posterior, respectively, whereas both classes remained above 93% when only the polar-motion features were used (Supplementary Table S5). These results indicate that the undecomposed Cartesian flow features were adequate for describing the presence of abnormal motion but insufficient for resolving its rotational direction, whereas the tangential and radial components provided more discriminative cues for lateralized pathological classification.

Permutation feature-importance analysis further showed that the mean tangential velocity (*v_t__mean*) ranked highest in the global feature ranking, showing the largest drop in Macro-F1 after shuffling (Figure 7A). When evaluating the overall contribution by category, the polar-motion terms dominantly drove the model’s predictive power compared to base flow and geometric terms (Figure 7C). This pattern supports the central role of polar motion features in posterior-canal BPPV side discrimination. Moreover, despite the closely matched baseline Macro F1-score performance between the two sampling rates (Figure 7D), the feature-importance rankings were highly consistent between the 60 fps and 30 fps models (Spearman ρ = 0.943; Figure 7B). *v_t__mean* remained the most influential feature in both settings, further supporting the stability of the polar-motion representation across sampling rates.

**Figure 7:**
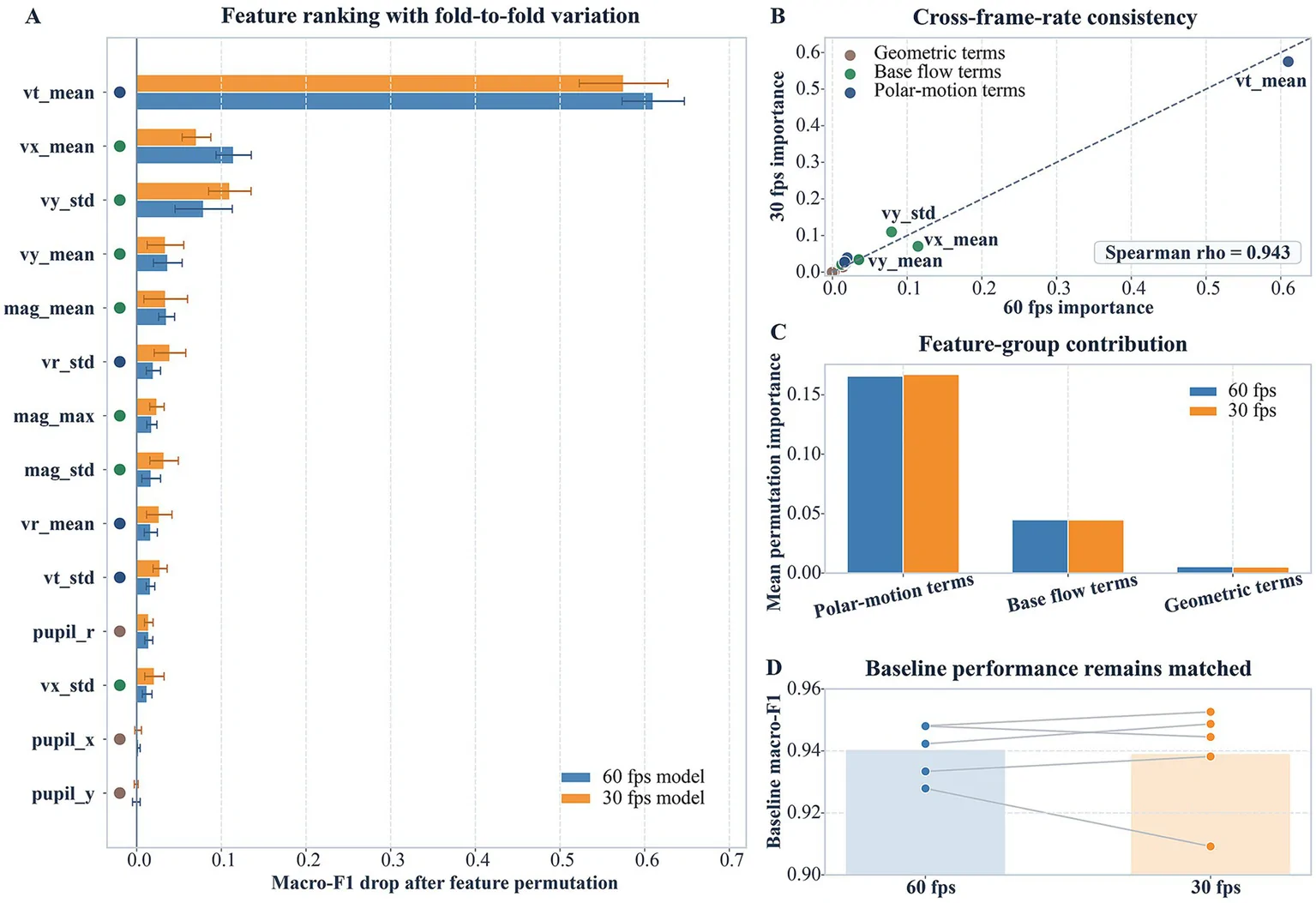
Effectiveness of feature decoupling analyzed via feature permutation importance for the 60 fps and 30 fps models. **(A)** Global feature ranking of the 14D features (measured by the drop in Macro F1-score after permutation). The mean tangential velocity (vt_mean) ranked highest in permutation importance, showing the largest drop in Macro-F1 after shuffling. **(B)** High cross-frame-rate consistency of feature importance distributions between the 60 fps and 30 fps models (Spearman ρ = 0.943). **(C)** Overall contribution of different feature groups (polar-motion terms, base flow terms, and geometric terms) to model predictions. **(D)** Matched baseline Macro F1-score performance between the 60 fps and 30 fps models.

### 3.4 Patient-level inference on the independent long-video test set

To assess generalizability under a more realistic clinical workflow, we evaluated the final 60 fps and 30 fps pipelines on an independent long-video test set comprising 45 Dix–Hallpike-related recordings from different patients, including 14 Normal, 14 Left Posterior, and 17 Right Posterior cases. All videos were successfully processed through feature extraction and patient-level inference, yielding a 100% processing success rate.

At 60 fps, the overall patient-level accuracy was 97.78% (44/45), as shown in Table 5. All 14 Normal videos were correctly classified, corresponding to a specificity of 100%, and all 14 Left Posterior videos were correctly detected, corresponding to a recall of 100%. Among the 17 Right Posterior videos, 16 were correctly identified and one was misclassified as Normal, yielding a recall of 94.12%. Importantly, the only patient-level error was a one-way false negative from Right Posterior to Normal; no Normal case was misclassified as pathological.

**Table 5:** Patient-level inference performance on the independent long-video test set.

Metric	Result	Description
Overall accuracy	97.78%	44 of 45 long videos were correctly classified
Specificity for Normal	100.00%	14/14 Normal videos were correctly classified
Recall for Left Posterior	100.00%	14/14 Left Posterior videos were correctly detected
Recall for Right Posterior	94.12%	16/17 Right Posterior videos were correctly detected; 1 was missed as Normal

A detailed temporal profile of a correctly recognized Right Posterior case is presented in Figure 8, showcasing the system’s dynamic tracking capabilities. As shown in Figure 8A, the continuous predicted probabilities accurately capture the onset and transition of the nystagmus event, which are then converted into stable segment-level class labels through majority voting (Figure 8B). Importantly, the synchronized temporal profile of the blink ratio and maximum prediction confidence (Figure 8C) demonstrates the framework’s high resilience; even during periods of frequent blinking (gray bars), the system maintains high-confidence classification output and successfully implements decay-tail suppression during the final phases of recording. In addition, the dynamic visualization of this automated inference process for the three diagnostic categories is provided in Supplementary Videos S1–S3.

**Figure 8:**
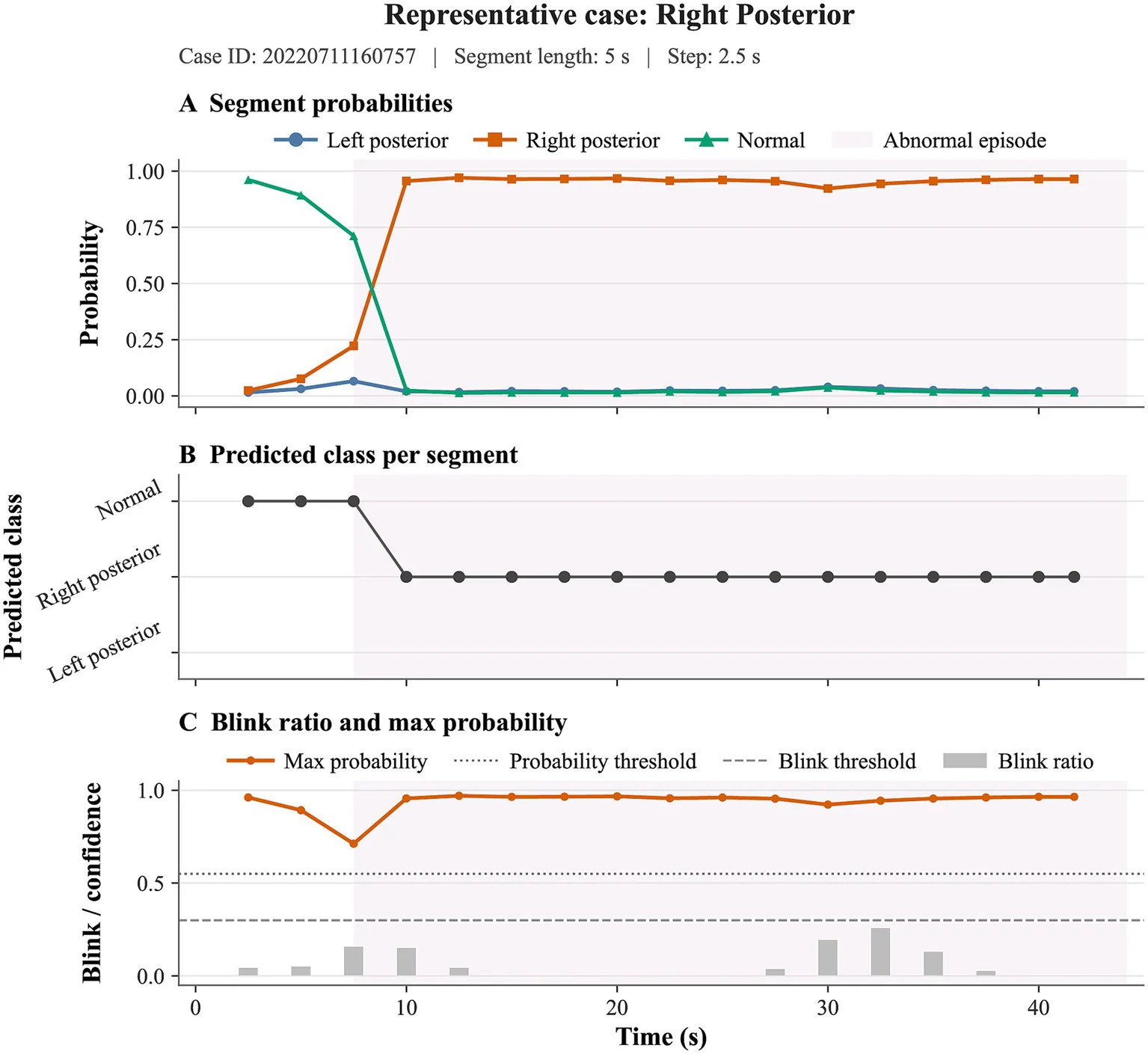
Representative case of patient-level inference and abnormal event reconstruction in the independent clinical long-video test set (Diagnosis: Right Posterior BPPV). **(A)** The temporal trajectory of the continuous segment-level three-class predicted probabilities over time (seconds). **(B)** The sequence of hard-label predicted classes per segment generated via probability thresholds and majority voting. **(C)** The temporal distribution of transient abnormal noise (Blink ratio, gray bars) and the system’s maximum prediction confidence (Max probability, solid line).

Further review of the misclassified case showed that the majority of its 15 sliding-window segments had been predicted as Normal, with an average maximum prediction probability of 0.8834. This pattern suggests that the error was more likely related to insufficient segment-level representation than to failure of the patient-level aggregation strategy.

At 30 fps, the model achieved 100% patient-level accuracy (45/45) on the same independent test set. The complete set of 45 long videos had a total duration of 28.8 min (1,725.4 s) and required approximately 16.7 min for end-to-end processing, representing nearly a 50% reduction in computational cost relative to the 60 fps baseline. These findings indicate that, in the present independent test cohort, the 30 fps configuration maintained excellent classification performance while improving inference efficiency.

### 3.5 Temporal localization and duration estimation of nystagmus events

As shown in Figure 9A, both the 60 fps and 30 fps pipelines achieved robust temporal localization of abnormal nystagmus events. Across the 31 annotated videos, the 60 fps pipeline achieved a mean temporal IoU of 0.899 and a hit rate of 96.8% (30/31), whereas the 30 fps pipeline achieved a comparable mean temporal IoU of 0.896 and a hit rate of 100% (31/31). The duration-ratio analysis further indicated that the predicted abnormal intervals closely approximated the manually annotated intervals, supporting the ability of the framework to estimate the temporal extent of induced nystagmus.

**Figure 9:**
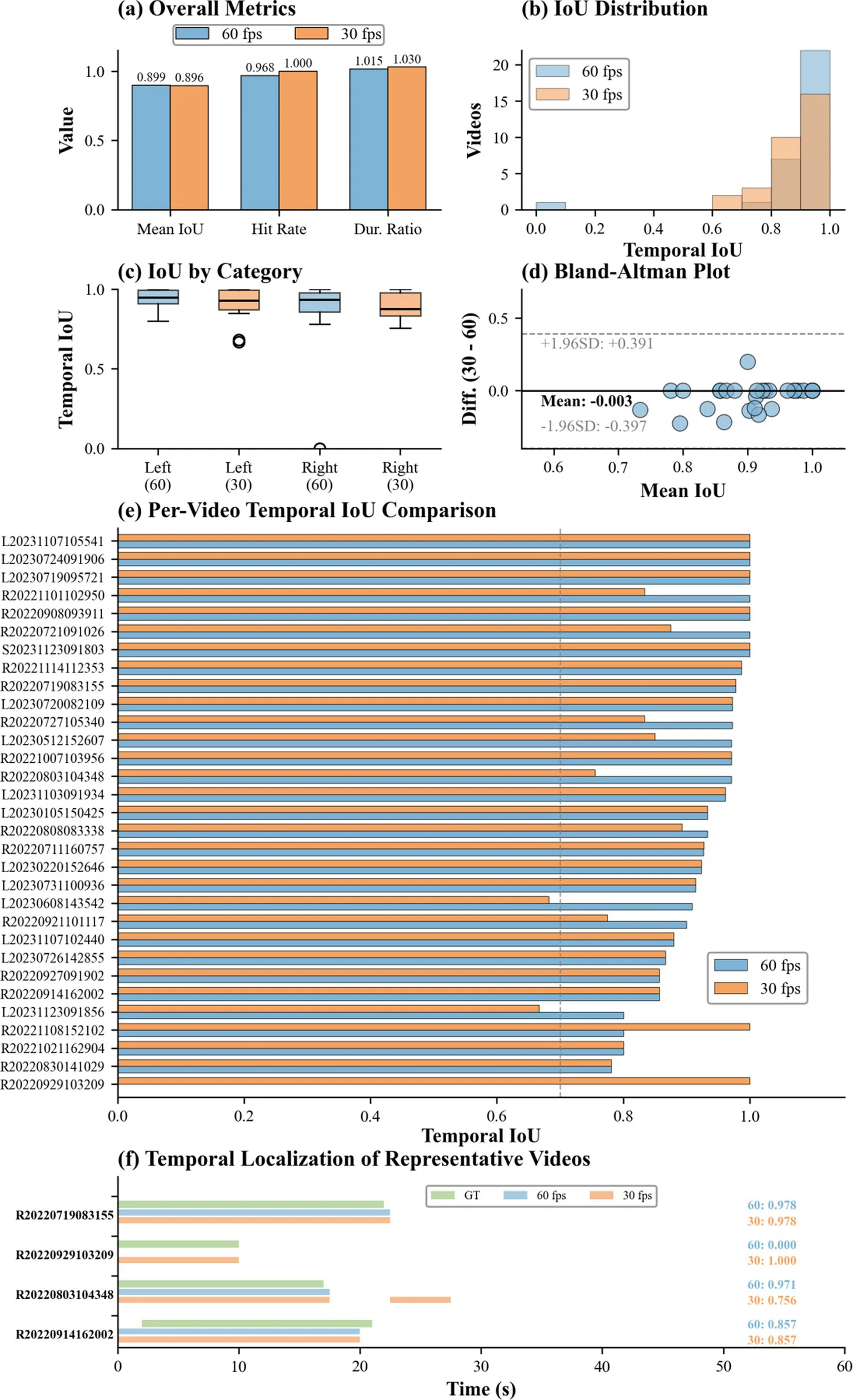
Temporal localization performance of abnormal nystagmus events under 60 fps and 30 fps inference. **(A)** Overall localization metrics. The 60 fps pipeline achieved a mean temporal IoU of 0.899 and a hit rate of 96.8% (30/31), while the 30 fps pipeline achieved a comparable mean IoU of 0.896 and a hit rate of 100% (31/31). **(B)** Distribution of temporal IoU values across 31 annotated videos. **(C)** Diagnosis-stratified temporal IoU for left and right posterior-canal BPPV. **(D)** Bland–Altman agreement between 60 fps and 30 fps temporal IoU measurements. **(E)** Per-video IoU comparison. **(F)** Representative temporal localization examples, with ground truth shown in green, 60 fps predictions in blue, and 30 fps predictions in orange.

The distribution of temporal IoU values further confirmed the robustness of event localization (Figure 9B). Most videos showed high overlap between the predicted and ground-truth intervals, and diagnosis-stratified analysis demonstrated consistently stable performance in both left posterior-canal and right posterior-canal BPPV cases (Figure 9C). Bland–Altman analysis showed close agreement between the 30 fps and 60 fps configurations, with a mean IoU difference of −0.003 and 95% limits of agreement ranging from −0.397 to +0.391 (Figure 9D). All annotated cases fell within the limits of agreement, indicating that downsampling from 60 fps to 30 fps did not materially affect temporal localization consistency.

Per-video comparison and representative examples are shown in Figure 9E and Figure 9F. The two frame-rate settings produced highly similar temporal IoU values in most videos. Notably, in the frame-rate-sensitive case R20220929103209, the 60 fps pipeline failed to retain the correct abnormal interval after post-processing, whereas the 30 fps pipeline correctly localized the right posterior-canal BPPV episode. One possible explanation is that downsampling increased the apparent inter-frame displacement of weak torsional eye movements, thereby making subtle nystagmus more distinguishable in the optical-flow-derived features. In contrast, at 60 fps, the smaller frame-to-frame displacement may be more easily affected by local tracking instability, blink-related artifacts, or confidence-based suppression during post-processing. Overall, these results indicate that the proposed framework can reliably detect abnormal nystagmus intervals and estimate event duration from long Dix–Hallpike recordings, while the 30 fps configuration may provide additional robustness in selected weak-nystagmus cases.

## 4 Discussion

In this study, we developed a physiology-guided and interpretable artificial intelligence framework for automated analysis of monocular infrared Dix–Hallpike videos. The framework was designed to classify recordings into right posterior-canal BPPV, left posterior-canal BPPV, and normal-negative recordings, while also localizing abnormal positional nystagmus intervals in long clinical recordings. By integrating deep learning-based pupil anchoring, anatomically constrained iris-ring masking, polar-flow decomposition, and temporal sequence modeling, the proposed method achieved stable segment-level classification in patient-level cross-validation and maintained high patient-level performance in independent long-video testing. Importantly, the framework did not rely solely on a black-box end-to-end prediction process. Instead, it transformed raw video motion into physiologically meaningful intermediate features, thereby providing a more transparent basis for clinical video review.

A major contribution of this work is the use of vestibular eye-movement physiology to guide the construction of interpretable video features. In posterior-canal BPPV, Dix–Hallpike-induced nystagmus is characterized by a torsional-upbeat pattern, and the direction of the torsional quick phase, described in a head-referenced frame by the movement of the upper pole of the eye toward the affected ear, provides essential information for left–right lateralization (28). Conventional Cartesian optical-flow descriptors may mix torsional movement with translational drift, vertical eye movement, eyelid motion, and image artifacts. In contrast, the proposed pupil-centered polar-flow decomposition explicitly separates motion into tangential and radial components around the pupil center. Because tangential flow directly reflects rotational motion around the pupil, it provides a physiologically meaningful representation of torsional nystagmus. The ablation and feature-importance analyses further supported this interpretation, as polar-motion features preserved most of the model performance and mean tangential velocity emerged as a highly influential feature. These findings suggest that the model primarily used clinically relevant torsional-motion information rather than nonspecific image-level cues.

This domain-knowledge-guided design is particularly relevant for explainable and interpretable AI in healthcare. Many deep learning systems achieve high performance but provide limited insight into why a specific prediction is made, which may restrict clinician trust and hinder real-world adoption. The present framework embeds interpretability into the feature construction process itself. Pupil localization defines the anatomical reference point, iris-ring masking focuses the analysis on the region where torsional motion is visually informative, and polar-flow decomposition converts dense motion fields into clinically understandable descriptors. Therefore, the explanation is not only generated after model prediction, but is partially built into the representation used by the model. This structure allows clinicians and researchers to relate the model output to recognizable vestibular eye-movement patterns.

Compared with previous automated nystagmus analysis approaches, the proposed method provides a clearer connection between video-derived signals, model prediction, and clinical reasoning. Traditional VNG-based or pupil-trajectory-based methods often depend on accurate pupil boundary fitting or stable center tracking, which may be affected by eyelid occlusion, blinking, infrared reflections, and transient image degradation. Purely end-to-end spatiotemporal models can capture complex video patterns, but their decision process is often difficult to interpret. Our approach uses deep learning-based pupil detection mainly as a robust spatial anchor and then extracts dense motion information from the iris-ring region. The resulting polar-motion features are subsequently modeled over time. This hybrid design preserves the flexibility of data-driven learning while maintaining an interpretable physiological representation of torsional eye movement.

The temporal localization component further improves the transparency and clinical usability of the framework. In routine Dix–Hallpike video review, a single video-level prediction is often insufficient. Clinicians may need to determine whether nystagmus is present, when it begins, how long it persists, and whether the predicted lateralization is temporally consistent. By aggregating segment-level predictions across long videos, the framework provides localized abnormal intervals that can be directly reviewed by clinicians. In this sense, temporal localization serves as a practical form of explanation: it links the final classification result to specific video segments rather than presenting only an isolated probability score. The high temporal agreement with clinician annotations suggests that the proposed method may support structured and efficient review of long, unedited clinical recordings.

The comparable performance of the 30 fps and 60 fps configurations also has practical implications. Lower-frame-rate processing may reduce computational cost, storage burden, and deployment requirements, which is relevant for outpatient vestibular clinics, bedside assessment systems, and retrospective review of large video archives. One possible explanation is that moderate downsampling increases the apparent inter-frame displacement of subtle torsional movements, thereby improving the optical-flow-derived tangential signal, while also reducing high-frequency tracking jitter. However, this finding should be interpreted cautiously. The optimal frame-rate setting may depend on camera hardware, image quality, nystagmus intensity, examination protocol, and preprocessing strategy. Therefore, the 30 fps configuration should be regarded as a promising practical option rather than a universally optimal setting, and further validation across devices and institutions is needed.

From a clinical safety and ethical AI perspective, the proposed framework should be positioned as a clinician-in-the-loop decision-support tool rather than an autonomous analysis system. Its outputs, including class probabilities, predicted laterality, localized abnormal intervals, blink or quality-related information, and interpretable polar-motion features, are intended to support clinician review of Dix–Hallpike recordings (29). False-negative predictions may delay recognition of clinically relevant torsional nystagmus, whereas false-positive or wrong-laterality predictions may lead to inappropriate interpretation of the positional response. For this reason, final diagnostic and therapeutic decisions should remain under clinician responsibility. The value of the system lies in improving the standardization, efficiency, and traceability of video review, rather than replacing clinical judgment.

Several limitations should be acknowledged. First, this was a retrospective single-center study. Although patient-level cross-validation and an independent long-video test set were used, external validation across different institutions, infrared recording devices, patient populations, and examination protocols remains necessary (30). Second, the independent long-video test set was relatively small, and the high patient-level performance observed in this cohort should therefore be interpreted as preliminary. Although the inclusion of post-repositioning negative recordings was intended to reduce subject-specific visual confounding, very subtle residual eye movements below the threshold of clinical annotation cannot be completely excluded and may introduce some heterogeneity into the negative class. Third, the present study was based on monocular left-eye recordings, and simultaneous binocular recording may provide complementary information for characterizing the relative torsional and vertical components of positional nystagmus. Fourth, the model was specifically developed for a predefined three-class task comprising right posterior-canal BPPV, left posterior-canal BPPV, and nystagmus-negative Dix–Hallpike recordings, and its performance in broader positional nystagmus patterns remains to be established. In particular, the current framework is not designed to identify horizontal-canal conversion after repositioning or to independently determine treatment success across different BPPV subtypes. Post-repositioning changes in nystagmus pattern therefore require clinician reassessment and, when indicated, additional positional testing. Finally, although the framework provides physiologically meaningful feature-level and temporal explanations, their clinical utility has not yet been formally evaluated. Larger prospective multicenter studies are needed to assess robustness across variations in age, eye appearance, eyelid occlusion, nystagmus severity and duration, and video quality, as well as to determine whether the proposed outputs improve classification confidence, reduce review time, or support less experienced clinicians in real-world vestibular assessment.

In summary, this study demonstrates that a physiology-guided polar-flow representation can provide interpretable analysis of Dix–Hallpike-induced torsional nystagmus. By combining clinically meaningful motion decomposition with temporal sequence modeling and clinician-reviewable localization outputs, the proposed framework offers a transparent and practical approach for automated vestibular video analysis. Further prospective, multi-center, and workflow-oriented validation is warranted before routine clinical implementation.

## 5 Conclusion

In conclusion, this study presents a physiology-guided and interpretable framework for automated classification and temporal localization of posterior-canal BPPV-related nystagmus based on vestibulo-ocular reflex kinematics. By integrating deep learning-based spatial anchoring with anatomically informed polar-motion decomposition, the proposed framework separates torsional and radial motion components and achieves balanced performance across the two lateralized pathological classes. An additional advantage is its ability to localize the onset and offset of nystagmus episodes within long clinical recordings, which may facilitate more efficient neuro-otological review and quantitative assessment of positional vestibular responses. The strong contribution of tangential velocity further supports the physiological relevance of the proposed feature representation. Encouraging performance was also observed in the independent long-video cohort, although these findings should be regarded as preliminary given the relatively small, single-center sample. Further prospective multicenter validation across different infrared recording systems, patient populations, and examination protocols is required to establish the generalizability and clinical utility of the framework. With continued optimization and future implementation of stream-processing strategies, the proposed approach may provide a practical basis for computer-assisted analysis of Dix–Hallpike recordings in neuro-otological practice.

## Data Availability

The raw data supporting the conclusions of this article will be made available by the authors, without undue reservation.
