# Supplementary material for: Interpretable polar-flow decomposition for torsional nystagmus lateralization in posterior-canal BPPV
Source: Front Neurol. 2026 Oct 2;17:1963383. doi: 10.3389/fneur.2026.1963383 (PMC13671929; doi:10.3389/fneur.2026.1963383)
Supplement: Supplementary file 1 [file Supplementary_file_1.DOCX]

# *Supplementary Materials*

Supplementary Table S1. Class Distribution of the Dataset

| **Class** | **Number of Patients** | **Number of Segments** | **Percentage (%)** |
| --- | --- | --- | --- |
| Left Posterior | 82 | 1,630 | 26.18 |
| Right Posterior | 83 | 1,631 | 26.20 |
| Normal | **97** | 2,965 | 47.62 |
| Total | 262 | 6,226 | 100.00 |

** The 2,965 normal-negative segments include recordings from 97 originally normal subjects and follow-up negative recordings from 81 benign paroxysmal positional vertigo (BPPV) subjects after successful treatment. All 262 subjects are independent individuals.*

Supplementary Table S2. Data Distribution of the Patient-Level Nested 5-Fold Cross-Validation

| **Fold** | **Training Segments** | **Validation Segments** | **Test Segments** |
| --- | --- | --- | --- |
| Fold 1 | 4,309 | 691 | 1,226 |
| Fold 2 | 4,279 | 827 | 1,120 |
| Fold 3 | 4,251 | 767 | 1,208 |
| Fold 4 | 4,146 | 709 | 1,371 |
| Fold 5 | 4,289 | 636 | 1,301 |

Supplementary Table S3. Definition of the 14-Dimensional Optical Flow Motion Features

| **Feature Category** | **Feature Name** | **Description** |
| --- | --- | --- |
| Flow components | vx_mean, vx_std | Mean and standard deviation of horizontal optical flow |
|  | vy_mean, vy_std | Mean and standard deviation of vertical optical flow |
| Magnitude statistics | mag_mean, mag_std, mag_max | Mean, standard deviation, and maximum of optical flow magnitude |
| Tangential velocity | vt_mean, vt_std | Mean and standard deviation of tangential velocity (rotational motion) |
| Radial velocity | vr_mean, vr_std | Mean and standard deviation of radial velocity (expansion/contraction) |
| Geometric context | pupil_x, pupil_y, pupil_r | Pupil center coordinates and radius |

Supplementary Table S4. Hyperparameter Configuration for Model Training

| **Parameter** | **Configuration** |
| --- | --- |
| Optimizer | AdamW |
| Initial learning rate | 1×10⁻³ |
| Weight decay | 1×10⁻⁴ |
| Batch size | 64 |
| Maximum epochs | 100 (Early stopping) |
| Dropout | 0.4 |
| Hidden layer dimensionality | 128 |
| Number of GRU layers | 2 |
| Label smoothing | 0.05 |

Figure S1. Representative video frame examples used for patient-level long-video inference.

Figure S2. Training dynamics and validation performance of the You Only Look Once version 8 nano (YOLOv8n) pupil detection module. (A) Progression of the bounding box regression loss (Box loss) and mean average precision (mAP@0.5:0.95) over 100 training epochs, exhibiting rapid convergence. (B) Core validation metrics achieved at the best epoch (Epoch 85), with mAP@0.5 reaching 99.50%. (C) Precision-Recall (PR) curve and F1-Confidence curve on the validation set. (D) Representative detection results on test images, illustrating that the model robustly regresses the pupil bounding box under complex clinical conditions, including varying infrared lighting, diverse gaze directions, and severe eyelid occlusions.

**Supplementary Table S5. Fine-Grained Diagnostic Performance of Different Feature Subsets Across Three Classes**

| **Feature Subsets** | **Normal F1-Score (%)** | **Left Posterior F1-Score (%)** | **Right Posterior F1-Score (%)** |
| --- | --- | --- | --- |
| Full feature set (14D) | 94.87 ± 1.23 | 93.73 ± 1.05 | 93.37 ± 0.94 |
| Polar-motion only (4D) | 94.33 ± 1.00 | 93.29 ± 1.46 | 93.10 ± 1.08 |
| Basic optical flow only (7D) | 93.88 ± 1.41 | 82.91 ± 2.40 | 83.32 ± 1.69 |
| Geometric features only (3D) | 79.69 ± 3.99 | 54.06 ± 5.17 | 54.12 ± 4.60 |

**Supplementary Videos**

**Video UI Legend**

To facilitate a comprehensive understanding, all supplementary videos utilize a standardized multi-panel visualization interface:

- **Top Row:** From left to right, the panels display the raw clinical infrared nystagmus video; the **anatomical mask** alongside the decoupled **polar dense optical flow field**; and the real-time pupil localization and dynamic iris-ring tracking powered by YOLOv8n.
- **Middle Row:** Four real-time waveforms illustrate the motion features extracted from a 5-second sliding window, including horizontal/vertical flow and the explicitly decoupled physiological features—**tangential velocity (*v_t_*, representing rotational motion)** and radial velocity (*v_r_*). **The red vertical lines overlaid on the waveforms indicate the automated blink and noise masks** used to suppress transient artifacts.
- **Bottom Row:** This panel presents the current segment index, the real-time predicted class with its maximum probability, and a visual bar chart showing the probability distribution across the three diagnostic categories (Left Posterior, Right Posterior, and Normal).

**Video S1. Visualization of automated long-video inference for a Left Posterior pc-BPPV case.** This video demonstrates the end-to-end diagnostic process for a typical Left pc-BPPV patient. Through polar motion decoupling, the system captures significant fluctuations in tangential velocity characteristic of torsional nystagmus, maintaining a consistently high probability for the "Left Posterior" class. The video highlights the framework's ability to precisely localize pathological episodes within a long recording.

**Video S2. Visualization of automated long-video inference for a Right Posterior pc-BPPV case.** This video showcases the inference process for a Right pc-BPPV case, emphasizing the system's precision in discriminating rotational direction (dextrorotation). Despite the presence of minor head movements and partial eyelid occlusion, the anatomical mask and polar-flow extraction ensure stable feature representation and balanced lateralization performance.

**Video S3. Visualization of automated long-video inference for a Normal-negative case.** This video illustrates the framework’s robustness in a normal-negative recording. It specifically highlights the system's resilience against non-pathological eye movements, such as physiological fixational microsaccades and frequent blinking. The **red mask lines** effectively demonstrate how artifacts are filtered out to maintain a stable "Normal" diagnosis with high confidence.
